# 745 Evaluating Nurses' Perceptions of Code Cart Competency

**DOI:** 10.1093/jbcr/irac012.298

**Published:** 2022-03-23

**Authors:** Sarah A Folliard, Alicia K Grubbs, Robel Beyene, Kelly Doyle, Stephen Gondek, Miranda Young

**Affiliations:** Vanderbilt University Medical Center, Mount Juliet, Tennessee; Vanderbilt University Medical Center, Hendersonville, Tennessee; Vanderbilt University Medical Center, Nashville, Tennessee; Vanderbilt University Medical Center, Nashville, Tennessee; Vanderbilt University Medical Center, Nashville, Tennessee; Vanderbilt, Spring hill, Tennessee

## Abstract

**Introduction:**

This study was conducted to evaluate critical care nurses' perceptions of competency when using the code cart during Advance Cardiac Life Support (ACLS) resuscitation. During post-resuscitation debrief, it was noted that nurses reported lack of familiarity with using the code cart and could not quickly locate items when needed.

**Methods:**

Using an email distribution list sent to nurses employed in the Burn Center, a pre-survey was conducted to evaluate baseline competency levels and provide the opportunity to self report level of familiarity with the code cart and the roles needed to conduct effective ACLS resuscitation. Following the pre-survey, various educational opportunities were offered to the nursing staff including written and hands-on activities in a low-stress setting. These activities were offered to day and night shift nurses on several occasions, and there was no limit to how many times a nurse could participate. Following 3 months of targeted educational intervention, a post-survey was conducted to re-evaluate the nurses' perceived comfort level with using the code cart during ACLS resuscitation.

**Results:**

The average years of nursing experience among participants was 3-4, with only two participants reporting 5 or more years of critical care experience. The nurses reported an increase in self-reported confidence level with using the code cart. The most common educational intervention that nurses felt would increase their comfort levels with using the crash cart were more frequent simulation scenarios (ie: mock codes), and hands-on training with open crash carts during in a low stress setting.

**Conclusions:**

Because ACLS resuscitation is a stressful, chaotic event, it is not feasible for nurses to learn "in the moment." Critical care nurses in the Burn Center are trained on resuscitation and are required to maintain ACLS certification, but this is not enough training for them to feel comfortable with their responsibilities during a code. After identifying that nurses were not confident in being able to work the crash cart and could not find necessary items when asked to do so during an emergency, a plan for additional hands-on education in a low-stress setting was developed. Using a pre/post survey, the multi-faceted educational opportunities were evaluated to see if they would impact the nurses' self-reported perceptions of code cart competency. Results indicated that not only were the educational interventions successful, but the nurses would like further opportunities for ongoing participation to maintain their level of comfort with the crash cart.

